# Radiomics-Guided Deep Learning Networks Classify Differential Diagnosis of Parkinsonism

**DOI:** 10.3390/brainsci14070680

**Published:** 2024-07-04

**Authors:** Ronghua Ling, Min Wang, Jiaying Lu, Shaoyou Wu, Ping Wu, Jingjie Ge, Luyao Wang, Yingqian Liu, Juanjuan Jiang, Kuangyu Shi, Zhuangzhi Yan, Chuantao Zuo, Jiehui Jiang

**Affiliations:** 1 School of Communication and Information Engineering, Shanghai University, Shanghai 200444, China; lingrh@sumhs.edu.cn; 2 School of Medical Imaging, Shanghai University of Medicine & Health Science, Shanghai 201318, China; shirley2446@shu.edu.cn; 3 School of Life Sciences, Shanghai University, Shanghai 200444, Chinajiangjiehui@shu.edu.cn (J.J.); 4 Department of Nuclear Medicine & PET Center, National Clinical Research Center for Aging and Medicine, & National Center for Neurological Disorders, Huashan Hospital, Fudan University, Shanghai 200437, China; 5 School of Electrical Engineering, Shandong University of Aeronautics, Binzhou 256601, China; 6 Department of Nuclear Medicine, Inselspital, Bern University Hospital, University of Bern, 3010 Bern, Switzerland; 7 Computer Aided Medical Procedures, School of Computation, Information and Technology, Technical University of Munich, 85748 Munich, Germany

**Keywords:** radiomics, deep learning, Parkinsonian, brain PET imaging, glucose metabolism

## Abstract

The differential diagnosis between atypical Parkinsonian syndromes may be challenging and critical. We aimed to proposed a radiomics-guided deep learning (DL) model to discover interpretable DL features and further verify the proposed model through the differential diagnosis of Parkinsonian syndromes. We recruited 1495 subjects for ^18^F-fluorodeoxyglucose positron emission tomography (^18^F-FDG PET) scanning, including 220 healthy controls and 1275 patients diagnosed with idiopathic Parkinson’s disease (IPD), multiple system atrophy (MSA), or progressive supranuclear palsy (PSP). Baseline radiomics and two DL models were developed and tested for the Parkinsonian diagnosis. The DL latent features were extracted from the last layer and subsequently guided by radiomics. The radiomics-guided DL model outperformed the baseline radiomics approach, suggesting the effectiveness of the DL approach. DenseNet showed the best diagnosis ability (sensitivity: 95.7%, 90.1%, and 91.2% for IPD, MSA, and PSP, respectively) using retained DL features in the test dataset. The retained DL latent features were significantly associated with radiomics features and could be interpreted through biological explanations of handcrafted radiomics features. The radiomics-guided DL model offers interpretable high-level abstract information for differential diagnosis of Parkinsonian disorders and holds considerable promise for personalized disease monitoring.

## 1. Introduction

Atypical Parkinsonian syndrome is a group of diseases on the progressive neurodegenerative spectrum that affects the nervous system and the parts of the motor system controlled by nerves [1,2]. Clinical differential diagnosis between atypical Parkinsonian syndromes can be challenging and critical due to their potential to lead to vastly different Parkinsonian disorders [3,4,5]. Patients with idiopathic Parkinson’s disease (IPD) exhibit a clinical phenotype primarily characterized by parkinsonism, which can be asymmetric and responsive to levodopa. This phenotype strongly resembles the clinical syndromes seen in patients with multiple system atrophy (MSA) and progressive supranuclear palsy (PSP) [6,7]. Accurate and sensitive biomarkers for the differential diagnosis of Parkinsonian syndromes allow us to pursue more aggressive therapeutic strategies for individual patients.

Neuroimaging, particularly positron emission tomography (PET) with ^18^F-fluorodeoxyglucose (FDG) tracing, has been widely used to discover a wide spectrum of glucose metabolism pathological abnormalities [8,9,10]. Previous studies proposed various approaches based on visual evaluation and metabolic pattern analyses to develop more aggressive tools for the differential diagnosis of Parkinsonian disorders [11]. In particular, the metabolic disease’s pattern has been successfully demonstrated as a proper surrogate for differential Parkinsonian diagnosis [12,13,14,15]. However, these pattern analyses employ imaging data transformed into vector features and discover hyper- or hypo-metabolism brain regions, hence underutilizing the information of various dimension hierarchies. Meanwhile, recent studies also employed radiomics analysis into PET images and extracted high-dimensional and multi-hierarchical features for designing classification or prognostic models [16,17,18]. Abnormal metabolic regions are quantified with obvious features, typically focusing on local uptake heterogeneity or hyper- or hypo-metabolism boundaries. Nevertheless, a significant drawback is that radiomics features are often defined by mathematically or semantically handcrafted characteristics based on established expert knowledge. Although an increasing number of radiomics features are being proposed, abstract features derived from PET images seldom capture the full scope of information.

Recently, the emergence of artificial intelligence has shown a meaningful breakthrough and impressive progress owing to the advances in computing power and big data [19,20,21]. Previous studies have demonstrated that deep learning (DL) based on PET brain imaging may discover the wide spectrum of physiological changes and the metabolic differences between different Parkinsonian disorders and provide comparative or even superior diagnostic performance [3,22,23]. Previous studies had achieved sensitivities of 98.1%, 88.5%, and 84.5% and specificities of 90.0%, 99.2%, and 97.8%, respectively, for the diagnosis of IPD, MSA, and PSP [3,22]. However, the difficulty of interpretation and the absence of well-rooted explanations about the deep learning model limits its clinical application. However, some approaches such as gradient-weighted class activation mapping provide limited explanations, which focus on the highlighted brain regions contributing to the deep learning models [24,25]. The features derived from latent layers or other layers are difficult to link with the underlying pathology. Moreover, the more interpretable radiomics approach may assist the DL model by enhancing its interpretability.

Thus, we aimed to propose a radiomics-guided DL model to discover interpretable DL features from PET images and further verify the proposed model through the differential diagnosis of Parkinsonian syndromes. We hypothesized that the radiomics-guided DL model may capture the complex glucose metabolic changes and abstract semi-supervised features associated with different Parkinsonian syndromes.

## 2. Materials and Methods

### 2.1. Participants

We recruited 1495 subjects who underwent FDG PET scanning, including 220 healthy controls and a total of 1275 Parkinsonian patients with IPD, MSA, and PSP. All subjects were enrolled from the Huashan Parkinsonian PET Imaging (HPPI) Database established by Department of Nuclear Medicine & PET Center, Huashan Hospital, Fudan University. All procedures involving human participants were conducted in accordance with the ethical standards of the institutional and/or national research committee and the 1964 Declaration of Helsinki and its later amendments or comparable ethical standards. This study’s data analysis and ethical approval were granted by the institutional review board of Huashan Hospital (identifier: KY2011-174, Date: 24 August 2011), and informed consent was obtained from all subjects.

**Figure 1:**
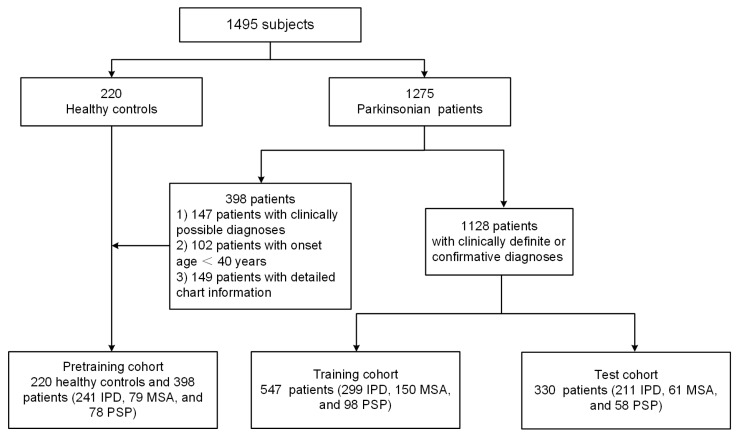
Flow of subjects through this study.

We investigated three independent cohorts of these subjects to develop and assess our proposed radiomics-guided DL model, as previously described [22]. In brief, the pretraining cohort consisted of 220 healthy controls and 398 Parkinsonian patients (241 IPD, 79 MSA, and 78 PSP) with clinically possible diagnoses, or with onset age < 40 years, or without detailed chart information. The pretraining cohort was used for preliminary training for the exclusion of non-Parkinsonian subjects. The training cohort contained 547 Parkinsonian patients (299 IPD, 150 MSA, and 98 PSP) with a clinically definite diagnosis, which was used to fine-tune the preliminary DL model. The test cohort consisted of 330 Parkinsonian patients (211 IPD, 61 MSA, and 58 PSP) with a clinically confirmative diagnosis, which was used to evaluate the prediction performance. A detailed eligibility profile of these subjects is shown in Figure 1.

Patients with a history of structural brain abnormalities were excluded. All patients with Parkinsonian diagnosis satisfied the diagnostic criteria according to the latest clinical criteria [26,27,28]. Patients were assessed by movement disorder specialists based on their return visits (at least once) after PET examination and were further stratified into three clinical typings termed as clinically possible, clinically definite, and clinically confirmative diagnoses analogous to that described previously [22].

### 2.2. PET Acquisition and Preprocessing

After attenuation correction using low-dose CT, the emission scan was acquired 60 min post-injection of approximately 185 MBq ± 18.5 of FDG and lasted 10 min (Siemens Biograph 64 HD PET/CT, Siemens Healthcare, Erlangen, Germany). PET images were reconstructed using the ordered subset expectation maximization (OSEM) method. FDG PET images were spatially normalized to the PET brain template in the Montreal Neurological Institute (MNI) brain space using SPM12 software (Wellcome Department of Imaging Neuroscience, Institute of Neurology, London, UK) implemented in Matlab 9.11.0 (Mathworks Inc., Sherborn, MA, USA). Finally, normalized PET images were smoothed with a three-dimensional Gaussian filter of 10 mm full width at half maximum (FWHM). Local glucose metabolic activity normalized to global activity was measured using the standardized uptake value ratio (SUVR).

### 2.3. Baseline Radiomics Analysis

FDG PET images in the training and test cohorts were used to extract radiomics features using the open-source PyRadiomics 3.1.0 software [29]. Cortical and subcortical regions of interest (ROIs) were identified using the automated anatomical labeling 3 (AAL3) [30,31] and PD25 atlases [32] and included the following 20 regions: frontal cortex, parietal cortex, occipital cortex, temporal cortex, globus pallidus, nucleus accumbens, ventral tegmental, locus coeruleus, raphe nucleus, dentate nucleus, substantia nigra, red nucleus, subthalamic nucleus, caudate, putamen, pallidum, thalamus, medulla oblongata, midbrain, and pons base. From each ROI, 107 radiomics features were extracted as shown in Supplementary Table S1. A total of 2140 radiomics features were measured, reflecting the shape, glucose uptake distribution, texture (gray-level co-occurrence matrix, GLCM; gray-level run-length matrix, GLRLM; gray-level size zone matrix, GLSZM), and margin characteristics (Neighborhood Gray-Tone Difference Matrix, NGTDM) from the above 20 ROIs. Each radiomics feature was normalized using Z-scores, calculated from the mean and standard deviation of the feature values within the training cohort.

We employed multinomial logistic regression (LR) with a one-vs.-all strategy in the training cohort to select radiomics features for constructing the baseline radiomics model. This model integrates selected radiomics features to classify different Parkinsonian disorders. The optimal radiomics feature set and coefficients were determined using nested five-fold cross-validation and the least absolute shrinkage and selection operator (LASSO) in the training dataset. In detail, 80% of the training cohort was used for model training, and the remaining 20% was used as a validation dataset to test model parameters. The test cohort was used to evaluate the diagnostic performance of the proposed radiomics model. Sensitivity, specificity, positive predictive value (PPV), and negative predictive value (NPV) were assessed.

### 2.4. Radiomics-Guided DL Model

**Figure 2:**
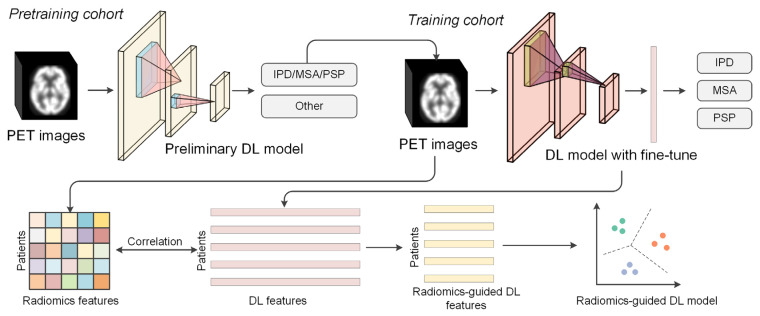
An illustration of the proposed radiomics-guided deep learning framework.

Figure 2 shows the graphical description of training the DL model and selecting candidate DL features guided by radiomics features for the differential diagnosis of Parkinsonian disorders. To extract abstract semi-supervised information on Parkinsonian disorders, the FDG PET images in the pretraining cohort were fed to a 3-dimensional residual network (ResNet) and Dense network for preliminary training for the exclusion of non- Parkinsonian subjects. Both deep learning models have demonstrated good classification performance in prior medical imaging studies, making them suitable as exemplar models for in-depth research [33,34,35].

We first pretrained the 121-layered DenseNet and 18-layered ResNet in the pretraining cohort for Parkinsonian diagnosis. The ResNet consisted of 18 layers, organized into eight residual blocks, each containing 2 convolutional layers. The architecture included an initial convolutional layer followed by a max pooling layer. Each residual block comprised two convolutional layers with a ReLU activation function and batch normalization. The Dense architecture included 121 layers, consisting of dense blocks, where each layer was connected to every other layer in a feed-forward fashion. The network comprised four dense blocks separated by transition layers that included convolution and pooling operations. Each dense block contained multiple layers with batch normalization, ReLU activation, and convolutional operations. Subsequently, we incorporated feature extraction from the pretrained models for IPD, MSA, and PSP diagnosis in the training and test cohort. The final layers of the pretrained networks were removed, allowing the networks to serve as feature extractors. By inputting 96 × 96 × 96-sized images, we obtained 65,280 and 122,800 DL features from the last convolutional layer of ResNet and DenseNet, respectively, as the latent variables representing the DL features of the metabolic abnormality patch.

All DL features were Z-score normalized using the mean and standard deviation of the training cohort before further processing. To select and filter the candidate DL features, we measured the correlation between all pairs of radiomics and DL features, retaining those with an absolute Pearson’s correlation greater than 0.4 and a Bonferroni-corrected *p*-value less than 0.05 in the training cohort. Our goal was to identify and explain key DL features in the latent layer. We then developed a compact radiomics-guided DL model by correlating radiomics features with DL features. The selected DL features were used in the multinomial logistic regression (LR) model. This multinomial LR model based on DL features was applied to the test cohort to evaluate its predictive performance.

### 2.5. Statistics Analysis

We used the one-way analysis of variance (ANOVA) with Bonferroni multiple comparisons to compare continuous-valued information and the Chi-square test to compare gender information. The multinomial LR model was employed to measure the classification performance of the retained features. And the Odds Ratio (OR) of the LR model was used to assess the effect of independent features on the differential diagnosis of Parkinsonism. All statistical analyses were conducted using SPSS Statistics 24. Two-sided *p* values of less than 0.05 were considered significant.

## 3. Results

### 3.1. Demographics

The clinical and demographic information of patients is shown in Table 1. The PSP patients were relatively older than other groups, with statistical significance (*p* < 0.001). The neuropsychological scores of Parkinsonian disorders differed significantly (*p* < 0.001), with lower Hoehn and Yahr stage scores and Unified Parkinson’s Disease Rating Scale (UPDRS) scores in IPD patients. There was no significant group difference for gender (all *p* > 0.05).

### 3.2. Baseline Radiomics Model

Radiomics analysis was used to calculate a total of 2140 features for each subject and was further regarded as a benchmark model. We used the cross-validation and LASSO method to penalize the differential diagnosis model. After the feature selection in the randomized cross-validations in 500 iterations, 35 top features were selected as the optimal feature set. Table S2 shows the differential diagnosis performance using radiomics for both training and test datasets. The sensitivity in the test dataset was 79.7% for IPD, 55.7% for MSA, and 56.1% for PSP. This modest diagnosis performance of radiomics indicates that metabolism abnormalities of MSA/PSP are less distinct compared to other atypical Parkinsonian syndromes. This suggests the need for higher-dimensional features to accurately identify these subtle metabolism dysfunctions.

### 3.3. Radiomics-Guided DL Model

Table S3 summarizes the classification performances of the initial ResNet and Dense network. Relative to the baseline radiomics model, these two networks significantly enhanced the accuracy of differential diagnosis among Parkinson’s disease subtypes, particularly for the MSA and PSP phenotype, achieving sensitivity exceeding 85%. Through the first correlation filtering of DL latent features, we retained 48 and 73 DL features for ResNet and DenseNet with corresponding Bonferroni-corrected *r*-values over 0.4. By sorting the LASSO regression weight coefficients, we retained four DL latent features from each model to construct the multinomial LR models for ResNet and DenseNet. The classification performances of these radiomics-guided DL models are shown in Table 2. The diagnosis ability using radiomics-guided DL models across both training and test datasets surpassed that achieved solely through radiomics, suggesting the superior efficacy of the DL approach. DenseNet showed the best diagnosis ability (sensitivity: 95.7%, 90.1%, and 91.2%; specificity: 93.2%, 97.3%, and 97.4%; PPV: 96.2%, 88.7%, and 88.1%; NPV: 92.4%, 97.7%, and 98.1% for IPD, MSA, and PSP, respectively) for different Parkinsonian syndromes using retained DL features in the test dataset. Therefore, we conducted in-depth analyses of the DenseNet model in the subsequent analysis.

Table 3 shows the retained DL latent features of the radiomics-guided DenseNet model and its diagnosis ability for each Parkinsonian disorder. Our analysis revealed that DenseNet_Latent_13280 exhibited superior discrimination ability for MSA (OR = 15.6, 95% CI: 6.84–35.7, *p* < 0.001) and IPD (OR = 0.11, 95% CI = 0.06–0.20, *p* < 0.001), whereas DenseNet_Latent_15017 demonstrated higher discrimination ability for PSP (OR = 4.47, 95% CI: 1.63–12.3, *p* < 0.001).

### 3.4. Interpretation of the DL Features with Radiomics

To retain the DL features in a radiomics-guided manner and enhance their interpretability, we calculated the correlations between all possible DL features and radiomics features. Table 4 shows the DenseNet latent features associated with radiomics features. The Dense_Latent_13280 feature demonstrated significant associations with 97 radiomics features (*p* < 0.05). Among these, 10 radiomics features exhibited an absolute correlation coefficient exceeding 0.5, and the GLCM correlation in the ventral tegmental area showed the most significant association (*r* = 0.607, *p* < 0.001). Dense_Latent_2239 was significantly associated with 51 of the radiomics features, 5 of the radiomics features exhibited an absolute correlation coefficient exceeding 0.5, and the GLCM autocorrelation in the raphe nucleus area showed the most significant association (*r* = −0.555, *p* < 0.001). Dense_Latent_15017 was significantly associated with 74 of the radiomics features, 7 of the radiomics features exhibited an absolute correlation coefficient exceeding 0.5, and the GLRLM high gray-level run emphasis in the subthalamic nucleus area showed the most significant association (*r* = 0.582, *p* < 0.001). Dense_Latent_28203 was significantly associated with 51 of the radiomics features, 6 of the radiomics features exhibited an absolute correlation coefficient exceeding 0.5, and the GLCM difference entropy in the red nucleus area showed the most significant association (*r* = 0.542, *p* < 0.001).

The linkage between DL latent features and handcrafted radiomics features could interpret DL features and facilitate the tracking of high-level abstract information regarding abnormal metabolic activity. In the DenseNet model, the Dense_Latent_13280 feature could be interpreted as a composite of five GLCM features, three GLRLM features, one GLSZM feature, and one first-order feature. Each of these 10 radiomics features has well-defined mathematical definitions, thereby providing a degree of interpretability. Notably, these handcrafted features are based on biological principles, offering potential insights into their significance and relevance. The correlation of GLCM measures the linear dependency of gray-level values on their respective voxels in the GLCM and was the most contributing radiomics feature for Dense_Latent_13280. The other three DL latent features could also be similarly characterized. For example, the Dense_Latent_2239 feature could be interpreted as a combination of four GLCM features and one GLRLM feature. Moreover, radiomics features that were significantly associated with the DL latent features were mainly located in the red nucleus, raphe nucleus, midbrain, nucleus accumbens, and cingulate. And most of those DL latent features belonged to the GLCM features. The GLCM features mostly reflected the metabolic dysfunction of abnormal brain regions, thereby allowing for biological interpretation.

## 4. Discussion

Interpretable imaging-based biomarkers may contribute to the discovery of abnormal brain regions distinguishing between different Parkinsonian disorders, thereby enhancing our understanding of the underlying biology [36]. In this study, we investigated the interpretability of the radiomics-guided DL model using FDG-PET images and found that integrating DL with radiomics enables the exploration of brand-new metabolic features. This integration combines the strong classification capabilities of DL with the interpretability of radiomics, significantly enhancing the analysis of Parkinsonian syndromes.

A previous study suggested that an estimated 20–30% of patients initially diagnosed with PD undergo reclassification, following pathological examinations, as either MSA or PSP [37]. This misdiagnosis significantly impacts clinical care and research trials, resulting in inaccurate prognoses and variable therapeutic responses in both PD and atypical Parkinsonian syndromes [38]. Previous studies had employed the DL algorithm into FDG-PET images and dopamine transporter PET images, achieving a diagnostic accuracy of 98.6% [3,22]. However, the lack of interpretability or the limited interpretability of DL poses constraints on its clinical application and its utility in elucidating the mechanisms underlying Parkinsonian syndromes, particularly concerning the pathological distinctions between symptoms. Our results show that the radiomics-guided DL approach is feasible for the differential diagnosis of Parkinsonian syndromes.

In this study, we adopted ResNet and DenseNet, two widely used DL models in medical imaging, for initial training in the pretraining cohort to achieve Parkinsonian diagnosis. Subsequently, fine-tuning was conducted within the training cohort to accomplish the differential diagnosis of Parkinsonian syndromes. Our findings show that the DenseNet model demonstrates superior classification results. Our subsequent analyses concentrate solely on investigating it within DenseNet. The DL latent features were extracted from the final layer of each DL model, as this layer typically encapsulates the most abstract information. Correlation analysis of DL latent features and radiomics was employed for feature selection to mitigate the challenge posed by an excessive number of features and the difficulty in achieving interpretability. In the radiomics-guided DenseNet model, we observed four latent features, Dense_Latent_13280, Dense_Latent_2239, Dense_Latent_15017, and Dense_Latent_28203, showed absolute correlation coefficients exceeding 0.5 with 10, 5, 7, and 6 radiomics features, respectively. Each DL latent feature could be elucidated as a composite of various handcrafted radiomics features. Notably, radiomics features indicative of glucose metabolism emerged as predominant across all four DL features, affirming the significance of distinctive glucose metabolism patterns in the differential diagnosis of Parkinsonian disorders. In detail, the long run emphasis of GLRLM measures the distribution of long run lengths, the large area emphasis of GLSZM measures the distribution of large-area size zones, the correlation of GLCM measures the linear dependency of gray-level values to their respective voxels, difference variance of GLCM reflects the heterogeneity that places higher weights on differing intensity level pairs that deviate more from the mean, and the high gray-level run emphasis of GLRLM measures the concentration of high gray-level values in the image. These radiomics measure the intensity and texture information of SUVR within the specific region and further offer a comprehensive characterization of glucose hyper-/hypo- metabolism within the brain region. Moreover, our findings show that radiomic features significantly related to DL latent features are mainly located in the red nucleus, raphe nucleus, midbrain, nucleus accumbens, and cingulate. These findings are in line with previous studies showing that the MSA group exhibited pronounced hypometabolism in the putamen, pons, and cerebellum relative to both normal controls and the IPD group [39,40,41]. Moreover, PSP demonstrated notable hypometabolism in the caudate nucleus, thalamus, midbrain, and cingulate gyrus compared to normal controls, the IPD group, and the MSA group [42,43,44,45]. Detailed descriptions of deep learning features will help clinicians gain a deeper understanding of Parkinson’s disease and discover new, more effective imaging markers. The integration of deep learning and imaging omics will catalyze the development of effective tools for large-scale clinical imaging using artificial intelligence.

This study has several limitations. Our study employed two common DL models to perform the radiomics-guided DL approach. Other DL models may have higher diagnosis ability and provide comprehensive abstract information from FDG PET images, which requires further study. Future studies can refine and replace the classification model within this framework to enhance its overall effectiveness. Also, the FDG PET images utilized in this study were not subjected to partial volume correction (PVC) due to the absence of corresponding MRI images for some subjects. The integration of the morphometries from MRI and glucose metabolism from FDG PET images in any future study may further enhance the imaging-based biomarkers. Finally, we employed the handcrafted radiomics features to explain the DL latent features. We have only provided a basic physiological explanation for these radiomics features for FDG PET images. Offering a comprehensive physiological explanation for mathematically defined radiomic features reflecting glucose metabolism across different brain regions presents a significant challenge.

## 5. Conclusions

In this study, we investigated the radiomics-guided DL model to extract interpretable DL features from FDG PET images and further verify the proposed model through the differential diagnosis of Parkinsonian syndromes. The DL latent features were evaluated with the multinomial LR model, demonstrating that this approach outperformed the baseline radiomics approach. And the retained DL latent features were significantly associated with radiomics features and could be interpreted through biological explanations of handcrafted radiomics features. Hence, the radiomics-guided DL model offers novel high-level abstract information for the differential diagnosis of Parkinsonian disorders and holds considerable promise for personalized disease monitoring.

## Figures and Tables

**Table 1:** Clinical and demographic information of 1275 patients with different Parkinsonian disorders.

		IPD	MSA	PSP	*p* Value
Pretraining cohort	*n*	241	79	78	/
	Gender (M/F)	154/87	42/37	45/33	0.202
	Age	50.0 ± 15.2	57.5 ± 10.6	64.6 ± 8.6	<0.001
	Symptom duration (months)	/	/	/	/
	Hoehn and Yahr stage	/	/	/	/
	UPDRS	/	/		/
Training cohort	*n*	299	150	98	/
	Gender (M/F)	166/133	78/72	60/38	0.359
	Age	60.2 ± 8.5	57.8 ± 8.0	67.2 ± 8.0	<0.001
	Symptom duration (months)	45.3 ± 46.0	24.3 ± 17.1	35.0 ± 20.7	<0.001
	Hoehn and Yahr stage	2.2 ± 1.0	3.1 ± 0.8	3.2 ± 0.8	<0.001
	UPDRS	27.0 ± 14.3	30.6 ± 14.5	30.1 ± 13.5	0.02
Test cohort	*n*	211	61	58	/
	Gender (M/F)	130/81	32/29	39/19	0.241
	Age (years)	60.0 ± 7.6	58.5 ± 6.3	65.1 ± 6.6	<0.001
	Symptom duration (months)	39.0 ± 41.3	27.0 ± 20.1	34.1 ± 22.7	0.062
	Hoehn and Yahr stage	1.9 ± 0.9	2.9 ± 0.8	3.0 ± 0.8	<0.001
	UPDRS	22.8 ± 12.1	29.3 ± 14.4	26.8 ± 11.0	<0.001

Note: Unified Parkinson’s Disease Rating Scale (UPDRS); IPD: idiopathic Parkinson’s disease; MSA: multiple system atrophy; PSP: progressive supranuclear palsy.

**Table 2:** The classification performance of the radiomics-guided ResNet and Dense network model for the differential diagnosis of Parkinsonian disorders.

		Group	Sensitivity	Specificity	PPV	NPV
ResNet	Training cohort	IPD	0.959	0.931	0.944	0.950
		MSA	0.920	0.982	0.951	0.970
		PSP	0.979	0.997	0.989	0.995
	Test cohort	IPD	0.896	0.855	0.917	0.821
		MSA	0.819	0.973	0.877	0.959
		PSP	0.877	0.956	0.806	0.973
DenseNet	Training cohort	IPD	0.973	0.959	0.966	0.967
		MSA	0.966	0.992	0.979	0.987
		PSP	0.969	0.997	0.989	0.993
	Test cohort	IPD	0.957	0.932	0.962	0.924
		MSA	0.901	0.973	0.887	0.977
		PSP	0.912	0.974	0.881	0.981

**Table 3:** The logistic regression results of radiomics-based DenseNet model and the retained DL features.

Feature	Group	Standardized Coefficients (*β*)	Odds Ratio (OR, 95% CI)	*p*-Value
DenseNet_Latent_13280	IPD	−2.161	0.11 (0.06–0.20)	<0.001
	MSA	2.748	15.6 (6.84–35.7)	<0.001
	PSP	−0.218	0.81 (0.38–1.67)	0.561
DenseNet_Latent_2239	IPD	0.657	1.93 (0.94–3.94)	0.071
	MSA	−1.885	0.15 (0.05–0.44)	<0.001
	PSP	1.498	4.47 (1.63–12.3)	<0.001
DenseNet_Latent_15017	IPD	−2.095	0.12 (0.05–0.26)	<0.001
	MSA	0.746	2.11 (0.58–7.63)	0.255
	PSP	0.728	2.07 (0.95–4.52)	0.066
DenseNet_Latent_28203	IPD	−0.740	0.47 (0.25–0.88)	0.018
	MSA	−0.513	0.59 (0.18–1.95)	0.395
	PSP	1.108	3.03 (1.61–5.71)	<0.001

Note: IPD: idiopathic Parkinson’s disease; MSA: multiple system atrophy; PSP: progressive supranuclear palsy; CI: confidence interval.

**Table 4:** The correlation results between radiomics features and retained DL features.

DL Feature	Category	Radiomics Features	Brain Region Location	*r-*Value
Dense_Latent_13280				
	GLRLM	Long run emphasis	Frontal lobe	0.525
	GLSZM	Large area emphasis	Frontal lobe	0.519
	GLCM	Difference variance	Caudate	−0.513
	GLCM	Correlation	Ventral tegmental	0.607
	GLCM	Difference variance	Ventral tegmental	−0.586
	GLCM	Difference average	Red Nucleus	−0.536
	GLRLM	High gray-level run emphasis	Raphe nucleus	−0.507
	GLRLM	Long run high gray-level emphasis	Raphe nucleus	−0.502
	GLCM	Sum average	Nucleus accumbens	0.542
	First-order	Variance	Midbrain	−0.505
Dense_Latent_2239				
	GLRLM	Gray-Level Variance	Cingulate	0.527
	GLCM	Difference variance	Ventral tegmental	−0.551
	GLCM	Correlation	Red Nucleus	0.519
	GLCM	Autocorrelation	Raphe nucleus	−0.555
	GLCM	Sum average	Nucleus accumbens	0.534
Dense_Latent_15017				
	GLRLM	Gray-Level Variance	Cingulate	0.537
	GLRLM	High gray-level run emphasis	Subthalamic nucleus	0.582
	GLCM	Difference variance	Red Nucleus	−0.566
	GLCM	Autocorrelation	Raphe nucleus	−0.572
	GLRLM	High gray-level run emphasis	Raphe nucleus	−0.527
	GLCM	Correlation	Nucleus accumbens	0.516
	First-order	Variance	Midbrain	−0.503
Dense_Latent_28203				
	GLSZM	Large-area high gray-level emphasis	Cingulate	0.529
	GLCM	Difference entropy	Red nucleus	0.542
	GLCM	Cluster prominence	Raphe nucleus	−0.527
	GLSZM	High gray-level zone emphasis	Subthalamic nucleus	0.513
	GLRLM	Gray-Level Variance	Subthalamic nucleus	0.540
	First-order	Variance	Midbrain	−0.524

Note: GLCM: gray-level co-occurrence matrix; GLSZM: gray-level size zone matrix; GLRLM: gray-level run-length matrix.

## Data Availability

The data presented in this study are available on request from the corresponding author due to data legality reasons.

## Supplementary Materials

The following supporting information can be downloaded at https://www.mdpi.com/article/10.3390/brainsci14070680/s1: Table S1: The descriptions of all 107 radiomics features. Table S2: The classification performance of the radiomics model for the differential diagnosis of Parkinsonian disorders. Table S3: The classification performance of the initial ResNet and Dense network model for the differential diagnosis of Parkinsonian disorders.
