# Supplementary material for: Radiomics-Guided Deep Learning Networks Classify Differential Diagnosis of Parkinsonism
Source: Brain Sci. 2024 Jul 4;14(7):680. doi: 10.3390/brainsci14070680 (PMC11274493; doi:10.3390/brainsci14070680)
Supplement: Supplementary file 1 [file brainsci-14-00680-s001.zip › brainsci-3026840-supplementary.pdf]

Supplementary information of

Radiomics-guided deep learning networks classify Differential diagnosis of parkinsonism

Table S1. The descriptions about all 107 radiomics features.

Feature category	Label	Feature name
Shape feature	1	Elongation
	2	Flatness
	3	LeastAxisLength
	4	MajorAxisLength
	5	Maximum2DDiameterColumn
	6	Maximum2DDiameterRow
	7	Maximum2DDiameterSlice
	8	Maximum3DDiameter
	9	MeshVolume
	10	MinorAxisLength
	11	Sphericity
	12	SurfaceArea
	13	SurfaceVolumeRatio
	14	VoxelVolume
First order features	15	10Percentile
	16	90Percentile
	17	Energy
	18	Entropy
	19	InterquartileRange
	20	Kurtosis
	21	Maximum
	22	MeanAbsoluteDeviation
	23	Mean
	24	Median
	25	Minimum
	26	Range
	27	RobustMeanAbsoluteDeviation
	28	RootMeanSquared
	29	Skewness
	30	TotalEnergy
	31	Uniformity
	32	Variance
Gray Level Co-occurrence Matrix	33	Autocorrelation
	34	ClusterProminence
	35	ClusterShade
	36	ClusterTendency
	37	Contrast
	38	Correlation
	39	DifferenceAverage
	40	DifferenceEntropy
	41	DifferenceVariance
	42	Id
	43	Idm
	44	Idmn
	45	Idn
	46	Imc1
	47	Imc2
	48	InverseVariance

	49	JointAverage
	50	JointEnergy
	51	JointEntropy
	52	MCC
	53	MaximumProbability
	54	SumAverage
	55	SumEntropy
	56	SumSquares
Gray Level Dependence Matrix	57	DependenceEntropy
	58	DependenceNonUniformity
	59	DependenceNonUniformityNormalized
	60	DependenceVariance
	61	GrayLevelNonUniformity
	62	GrayLevelVariance
	63	HighGrayLevelEmphasis
	64	LargeDependenceEmphasis
	65	LargeDependenceHighGrayLevelEmphasis
	66	LargeDependenceLowGrayLevelEmphasis
	67	LowGrayLevelEmphasis
	68	SmallDependenceEmphasis
	69	SmallDependenceHighGrayLevelEmphasis
	70	SmallDependenceLowGrayLevelEmphasis
Gray Level Run Length Matrix	71	GrayLevelNonUniformity
	72	GrayLevelNonUniformityNormalized
	73	GrayLevelVariance
	74	HighGrayLevelRunEmphasis
	75	LongRunEmphasis
	76	LongRunHighGrayLevelEmphasis
	77	LongRunLowGrayLevelEmphasis
	78	LowGrayLevelRunEmphasis
	79	RunEntropy
	80	RunLengthNonUniformity
	81	RunLengthNonUniformityNormalized
	82	RunPercentage
	83	RunVariance
	84	ShortRunEmphasis
	85	ShortRunHighGrayLevelEmphasis
	86	ShortRunLowGrayLevelEmphasis
Gray Level Size Zone Matrix (GLSZM) Features	87	GrayLevelNonUniformity
	88	GrayLevelNonUniformityNormalized
	89	GrayLevelVariance
	90	HighGrayLevelZoneEmphasis
	91	LargeAreaEmphasis
	92	LargeAreaHighGrayLevelEmphasis
	93	LargeAreaLowGrayLevelEmphasis
	94	LowGrayLevelZoneEmphasis
	95	SizeZoneNonUniformity
	96	SizeZoneNonUniformityNormalized
	97	SmallAreaEmphasis
	98	SmallAreaHighGrayLevelEmphasis
	99	SmallAreaLowGrayLevelEmphasis
	100	ZoneEntropy
	101	ZonePercentage
	102	ZoneVariance
Neighbouring Gray Tone Difference Matrix (NGTDM) Features	103	Busyness
	104	Coarseness
	105	Complexity
	106	Contrast
	107	Strength

Table S2. The classification performance of radiomics model for differential diagnosis of parkinsonian disorders.

	Group	Sensitivity	Specificity	PPV	NPV
Training dataset	IPD	0.839	0.721	0.784	0.788
	MSA	0.706	0.924	0.779	0.892
	PSP	0.653	0.962	0.790	0.927
Test dataset	IPD	0.797	0.762	0.857	0.676
	MSA	0.557	0.903	0.566	0.900
	PSP	0.561	0.915	0.581	0.909

Note: PSP, progressive supranuclear palsy; IPD, idiopathic Parkinson's disease; MSA, multiple system atrophy; PPV: positive predictive value; NPV: negative predictive value.

Table S3. The classification performance of the initial ResNet and Dense network model for differential diagnosis of parkinsonian disorders.

		Group	Sensitivity	Specificity	PPV	NPV
ResNet	Training dataset	IPD	0.997	0.991	0.993	0.996
		MSA	0.993	0.997	0.993	0.997
		PSP	0.989	1	0.999	0.997
	Test dataset	IPD	0.885	0.988	0.947	0.974
		MSA	0.972	0.915	0.953	0.947
		PSP	0.859	0.971	0.859	0.971
Dense Network	Training dataset	IPD	0.993	0.997	0.993	0.997
		MSA	0.993	0.983	0.986	0.991
		PSP	0.969	0.997	0.989	0.993
	Test dataset	IPD	0.901	0.988	0.948	0.977
		MSA	0.966	0.923	0.957	0.939
		PSP	0.894	0.974	0.879	0.977
